# Chronic exposure to cigarette smoke leads to activation of p21 (RAC1)-activated kinase 6 (PAK6) in non-small cell lung cancer cells

**DOI:** 10.18632/oncotarget.11310

**Published:** 2016-08-16

**Authors:** Remya Raja, Nandini A. Sahasrabuddhe, Aneesha Radhakrishnan, Nazia Syed, Hitendra S. Solanki, Vinuth N. Puttamallesh, Sai A. Balaji, Vishalakshi Nanjappa, Keshava K. Datta, Niraj Babu, Santosh Renuse, Arun H. Patil, Evgeny Izumchenko, T.S. Keshava Prasad, Xiaofei Chang, Annapoorni Rangarajan, David Sidransky, Akhilesh Pandey, Harsha Gowda, Aditi Chatterjee

**Affiliations:** ^1^ Institute of Bioinformatics, International Tech Park, Bangalore, 560 066, India; ^2^ Department of Biochemistry and Molecular Biology, Pondicherry University, Puducherry, 605014, India; ^3^ School of Biotechnology, KIIT University, Bhubaneswar, Odisha, 751024, India; ^4^ Amrita School of Biotechnology, Amrita University, Kollam, 690 525, India; ^5^ Department of Molecular Reproduction, Development and Genetics, Indian Institute of Science, Bangalore, 560012, India; ^6^ Department of Otolaryngology-Head and Neck Surgery, Johns Hopkins University School of Medicine, Baltimore, Maryland, 21231, USA; ^7^ McKusick-Nathans Institute of Genetic Medicine, Baltimore, Maryland, 21205, USA; ^8^ Department of Biological Chemistry, Johns Hopkins University School of Medicine, Baltimore, Maryland, 21205, USA; ^9^ Department of Oncology, Johns Hopkins University School of Medicine, Baltimore, Maryland, 21205, USA; ^10^ Department of Pathology, Johns Hopkins University School of Medicine, Baltimore, Maryland, 21205, USA; ^11^ YU-IOB Center for Systems Biology and Molecular Medicine, Yenepoya University, Mangalore, 575018, India; ^12^ NIMHANS-IOB Proteomics and Bioinformatics Laboratory, Neurobiology Research Centre, National Institute of Mental Health and Neurosciences, Bangalore, 560029, India

**Keywords:** mass spectrometry, NSCLC, p21 (RAC1)-activated kinase 6, smoking

## Abstract

Epidemiological data clearly establishes cigarette smoking as one of the major cause for lung cancer worldwide. Recently, targeted therapy has become one of the most preferred modes of treatment for cancer. Though certain targeted therapies such as anti-EGFR are in clinical practice, they have shown limited success in lung cancer patients who are smokers. This demands discovery of alternative drug targets through systematic investigation of cigarette smoke-induced signaling mechanisms. To study the signaling events activated in response to cigarette smoke, we carried out SILAC-based phosphoproteomic analysis of H358 lung cancer cells chronically exposed to cigarette smoke. We identified 1,812 phosphosites, of which 278 phosphosites were hyperphosphorylated (≥ 3-fold) in H358 cells chronically exposed to cigarette smoke. Our data revealed hyperphosphorylation of S560 within the conserved kinase domain of PAK6. Activation of PAK6 is associated with various processes in cancer including metastasis. Mechanistic studies revealed that inhibition of PAK6 led to reduction in cell proliferation, migration and invasion of the cigarette smoke treated cells. Further, siRNA mediated silencing of PAK6 resulted in decreased invasive abilities in a panel of non-small cell lung cancer (NSCLC) cells. Consistently, mice bearing tumor xenograft showed reduced tumor growth upon treatment with PF-3758309 (group II PAK inhibitor). Immunohistochemical analysis revealed overexpression of PAK6 in 66.6% (52/78) of NSCLC cases in tissue microarrays. Taken together, our study indicates that PAK6 is a promising novel therapeutic target for NSCLC, especially in smokers.

## INTRODUCTION

Lung cancer accounts for ~19% of the cancer related deaths worldwide [1]. The 5 year survival rate is only 15% for patients diagnosed with lung cancer in the advanced stage [2]. Approximately 85% of the lung cancers are non-small cell lung cancer (NSCLC) [3]. Smoking is one of the major risk factors for NSCLC [4, 5]. NSCLC in smokers presents distinct molecular signatures compared to lung cancer from non-smokers [6, 7].

Anti-EGFR therapy such as gefitinib and erlotinib are currently in practice for treatment of NSCLC. Though, these drugs have shown marked the efficacy in non-smokers, smokers seem to be largely resistant [7]. Studies have shown that smokers acquire distinct EGFR mutations [8]. Smoke exposure also leads to aberrant phosphorylation of EGFR and downstream signaling that confers TKI-resistance in smokers [9]. Identification of novel agents that can act as therapeutic targets in such patients remains a challenge. In small percentage of NSCLC patients, targeted therapies that inhibit EML4-ALK or insulin-like growth factor 1 receptor (IGF-1R) are effective [10]. Dysregulation in other key signaling pathways such as PI3K/AKT/mTOR, Ras/Raf/MAPK and MET kinase have been reported as potential targets but are pending clinical validation. These observations accentuate the need for systematic investigation of alternative signaling pathways that are activated upon chronic exposure to smoke.

To understand the aberrant signaling in smokers in lung cancer, we developed a cell - based model, where lung cancer cell line H358, was chronically exposed to cigarette smoke condensate (CSC). Investigators have studied the effect of cigarette smoke at high dose and short exposure on lung cells [11–14]. However, clinical data has established that chronic cigarette smoke exposure and not acute exposure induce carcinogenesis. The above mentioned studies have elucidated perturbations in pathways such as EGFR in response to acute exposure to cigarette smoke. To date, there are limited studies addressing effect of chronic exposure of cigarette smoke in lung cells, even though smoking remains the primary risk factor for NSCLC.

Phosphoproteomics has emerged as a powerful tool to understand the global alterations in the signaling pathways [15, 16]. Further, for reliable quantitation of the phosphoproteome, SILAC, an *in vivo* proteome labeling technique has become a preferred choice [17]. We carried out high resolution mass spectrometry-based analysis to identify aberrantly activated signaling pathways in lung cancer by chronic cigarette smoke exposure. SILAC coupled with affinity-based enrichment of phosphopeptides was employed to identify dysregulated phosphosites upon chronic cigarette smoke exposure.

We identified a total of 3,624 phosphopeptides corresponding to 1,812 unique phosphosites and 1,086 proteins. Out of these, 278 phosphosites were found to be hyperphosphorylated (≥ 3-fold) in H358 cells exposed to cigarette smoke. The hyperphosphorylated proteins identified in our data includes p21 protein activated kinase 6 (PAK6) and epidermal growth factor receptor (EGFR) amongst others. In this study, we investigated the role of PAK6 in NSCLC. PAKs are involved in various processes including cell proliferation, survival, motility and are the major downstream effectors of Rho GTPase proteins including cdc42 and Rac1 [18, 19]. PAK4, 5 and 6 belong to the group II of PAKs which lack auto-inhibitory domain present in group I PAKs. Though previous reports have shown the overexpression of PAK6 in multiple cancers including prostate cancer, breast cancer and in hepatocellular carcinoma, there are limited studies investigating the signaling mechanism of PAK6 in cancer [20, 21]. In this study, we assessed the potential of PAK6 as a novel therapeutic target in NSCLC especially among smokers.

## RESULTS

### Chronic exposure to cigarette smoke leads to enhanced cell survival

**Figure 1:**
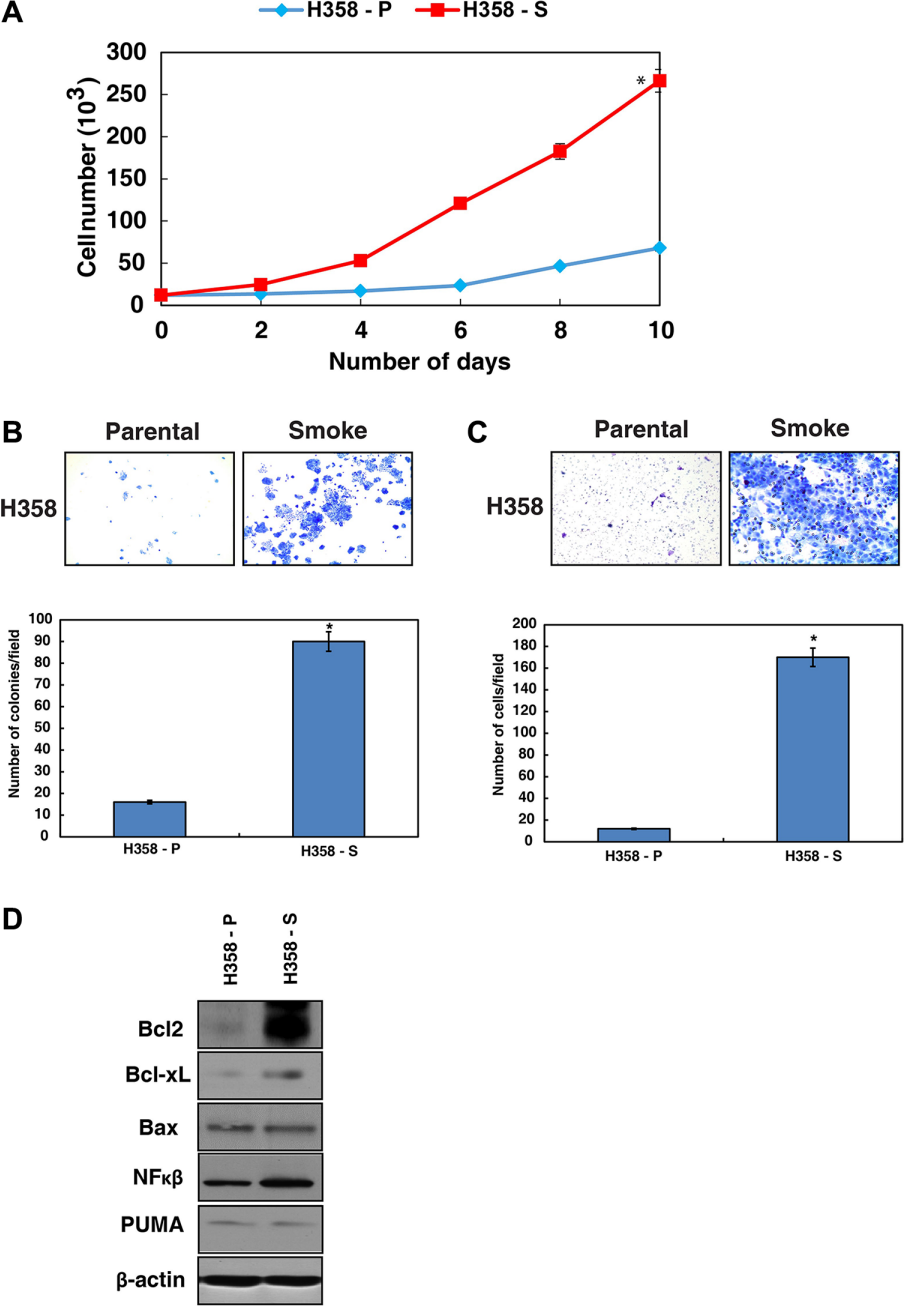
Chronic exposure to cigarette smoke leads to enhanced cell survival (**A**) Proliferation curve of H358-P and H358-S cells. (**B**) Colony forming ability of H358 cells after chronic treatment with CSC. (**C**) Invasive ability of H358 cells chronically treated with CSC. (**D**) Western blot analysis of the indicated proteins in the H358-P and H358-S cells. β-actin serves as a loading control.

To understand the effects of chronic cigarette smoke exposure in lung cancer cells, we developed a cell line model using H358 cells. H358 is a spontaneously immortalized lung cancer cell line derived from an *in situ* adenocarcinoma (earlier nomenclature - Bronchioalveolar carcinoma) and is a non to minimally invasive cell line. The cells lack the ability to grow in anchorage independent fashion was chosen for the study. These cells were exposed to CSC (0.1%) for 12 months and were designated as H358-S [22]. The H358 parental cells unexposed to smoke were referred as H358-P. During the course of chronic exposure, we observed alteration in both morphological (data not shown) and biological properties of the cells. We observed increased proliferation and colony formation with H358-S cells compared to the parental cells (Figure 1A and 1B). *In vitro* invasion assays using matrigel showed that the minimally-invasive H358 cells had acquired increased invasive property upon chronic cigarette smoke treatment and more than 80% of the cells had invaded the matrigel-coated PET membrane (Figure 1C). These results indicate an increase in both proliferative and invasive potential of H358 cells in response to chronic cigarette smoke exposure. It is established that genotoxic insults enable cancer cells escape cell death by regulating the expression of both pro- and anti-apoptotic proteins [23]. Since H358-S cells showed increased colony forming and invasive ability, we next examined the expression of BCL-2 family proteins in response to cigarette smoke. Western blot analysis revealed an increase in expression of both BCL-XL and BCL-2 in the H358-S cells compared to the parental cells. The transcription factor nuclear factor-kappaB (NF-kB) which can both suppress and promote apoptosis, showed an increased expression in the cigarette smoke treated cells. However, the expression levels of pro-apoptotic proteins like BAX and PUMA remained unchanged (Figure 1D). These results indicate that chronic exposure to cigarette smoke induces cellular transformation and increases the cell survival by modulating the expression of pro- and anti-apoptotic molecules.

### Chronic exposure to cigarette smoke induces widespread perturbation of signaling pathways

**Figure 2:**
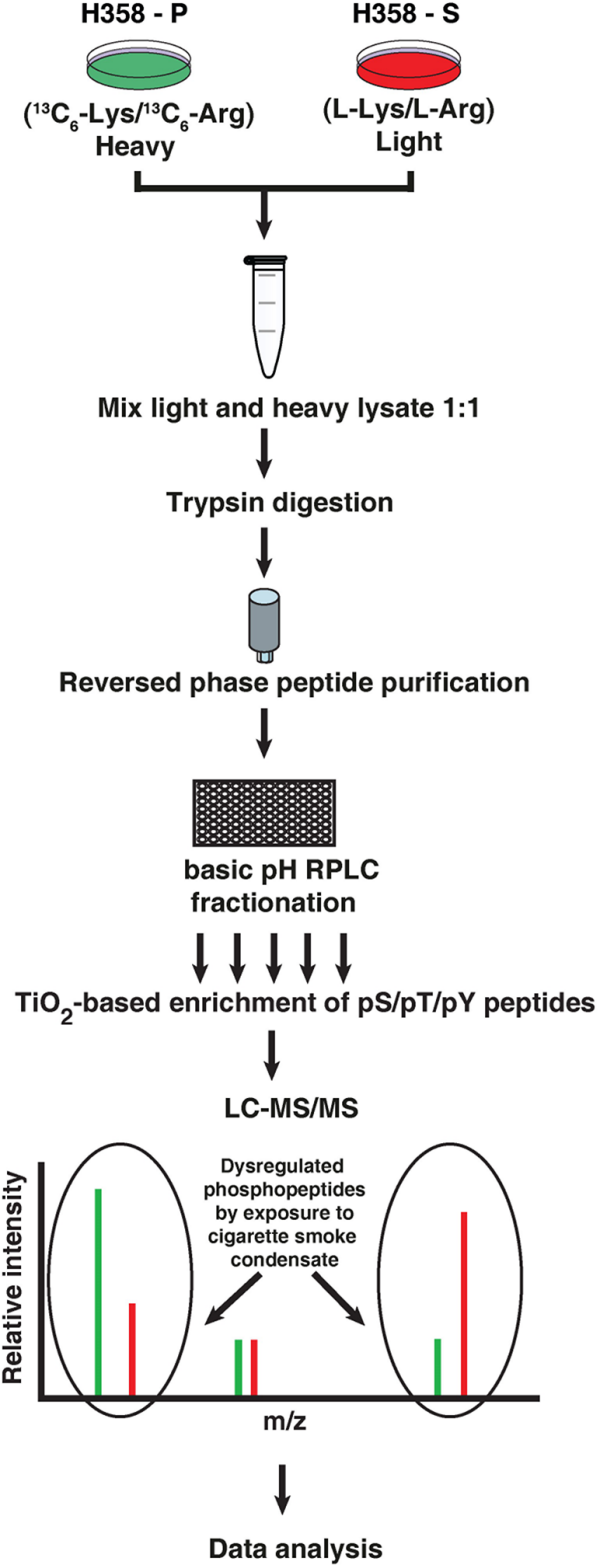
Schematic workflow followed to identify differentially phosphorylated proteome upon chronic exposure to cigarette smoke H358-P cells were grown in heavy media enriched with ^13^C_6_-Lysine/^13^C_6_-Arg while H358-S cells were cultured in light media (L-Lys/L-Arg). Equal amount of lysate from H358-P and H358-S cells were pooled and subjected to in-solution trypsin digestion, followed by reversed phase peptide purification and bRPLC fractionation. Phosphopeptide enrichment was carried out using titanium dioxide followed by mass spectrometry-based proteomic analysis to identify differentially phosphorylated proteins.

Since cigarette smoke led to an increase in the proliferation and invasive potential of the cells, we sought to study the altered signaling pathways in H358-S cells. We carried out SILAC-based phosphoproteomic analysis of H358-P and H358-S cells. The work flow is depicted in Figure 2. High resolution mass spectrometry-based analysis lead to identification of 1,812 phosphosites corresponding to 1,086 proteins amongst which 278 phosphosites were hyperphosphorylated while 125 were hypophosphorylated (≥ 3-fold) in H358-S cells. The partial list of hyperphosphorylated sites is shown in Table 1. The complete list of identified phosphopeptides is provided in Supplementary Table S1.

**Table 1:** Representative list of hyperphosphorylated phosphosites upon cigarette smoke exposure

	Protein	Gene symbol	Phosphopeptide	PhosphoRS	H358 CSC/H358
1	mitogen-activated protein kinase 14	*MAPK14*	HTDDEmTGyVATR	T(2): 0.0; T(7): 2.5; Y(9): 97.5; T(12): 0.0	12.6
2	GRB2-associated-binding protein 2	*GAB2*	NNTVIDELPFKsPITK	T(3): 0.0; S (12): 100.0; T(15): 0.0	8.7
3	epidermal growth factor receptor	*EGFR*	ELVEPLtPSGEAPNQALLR	T(7): 100.0; S(9): 0.0	7.5
4	hepatocyte nuclear factor 3-alpha	*FOXA1*	KDPSGASNPSADsPLHR	S(4): 0.0; S(7): 0.0; S(10): 0.0; S(13): 100.0	7.2
5	cyclin-dependent kinase inhibitor 1B	*CDKN1B*	VSNGsPSLER	S(2): 0.0; S(5): 98.6; S(7): 1.4	6.4
6	serine/threonine-protein kinase PAK 6	*PAK6*	sLVGTPYWMAPEVISR	S(1): 100.0; T(5): 0.0; Y(7): 0.0; S(15): 0.0	5.4
7	signal transducer and activator of transcription 3	*STAT3*	FIcVTPTTcSNTIDLPMsPR	T(5): 0.0; T(7): 0.0; T(8): 0.0; S(10): 0.0; T(12): 0.0; S(18): 100.0	4.6
8	ribosomal protein S6 kinase alpha-4	*RPS6KA4*	LEPVYSPPGsPPPGDPR	Y(5): 0.0; S(6): 0.0; S(10): 100.0	3.1
9	serine/threonine-protein kinase TAO3	*TAOK3*	NGPLNEsQEDEEDSEHGTSLNR	S(7): 100.0; S(14): 0.0; T(18): 0.0; S(19): 0.0	3.02
10	cyclin-dependent kinase 1	*CDK1*	IGEGTyGVVYK	T(5): 2.4; Y(6): 97.6; Y(10): 0.1	2.5

The table lists representative hyperphosphorylated phosphopeptide with corresponding protein name, gene symbol, phosphopeptide sequence, PhosphoRS score and H358-S/ H358-P fold-change.

Upon chronic cigarette smoke exposure, we observed hyperphosphorylation of some of the molecules known to be associated with lung cancer. We observed hyperphosphorylation of p27 (CDKN1B) at S10 (6.4-fold) which is associated with cell motility and inhibition of apoptosis [24]. MDM2, which is a known regulator of p53 was found to be hyperphosphorylated at S166. AKT-mediated phosphorylation of MDM2 at S166 is known to increase its interaction with p300, allowing MDM2-mediated ubiquitination and degradation of p53 leading to cancer progression [25]. These findings support our observation that cigarette smoke exposure increases the oncogenic potential of the lung cancer cells.

**Figure 3:**
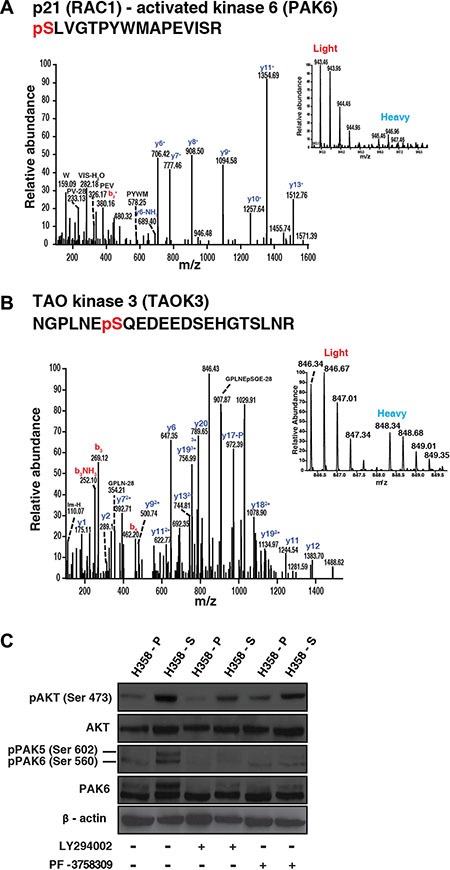
Representative MS/MS spectra of peptides of hyperphosphorylated proteins in H358-S cells (**A**) p21 activated kinase 6 (**B**) TAO kinase 3. (**C**) AKT mediates PAK6 phosphorylation in lung cancer cells exposed to cigarette smoke: H358-P and H358-S cells were treated with PF-3758309 and LY294002 respectively. Western blot was performed using phospho AKT, total AKT, phospho PAK6 and total PAK6 antibodies. β-actin was used as a loading control.

We also observed hyperphosphorylation of sites associated with activation of kinases which are known to play a key role in cancer progression such as EGF receptor (T693) (7.5-fold). Activation of EGF receptor has been reported in lung cancer patients who are smokers [9]. We also observed hyperphosphorylation of sites which are crucial to the activation of kinases such as TAOK3 (S324) (3-fold), MAPK14 (Y182) (12.5-fold), PAK6 (S560) (5.4-fold) and RPS6KA4 (S347) (3-fold). Amongst group II PAKs, increased expression of PAK4 is associated with poor prognosis in NSCLC [26, 27]. However, the expression and biological function of PAK6 in NSCLC is not known. We identified some of the upstream activators of PAK6 such as TAOK3 and MAPK14 which were previously reported to be involved in PAK6 signaling. Kaur et al., have reported activation of PAK6 by MAPK14 and PAK6 was found to be inhibited in presence of MAPK14 antagonist [28]. Also, in an independent study, TAOK3 was found to be an upstream regulator of MAPK14 in response to DNA damage [29]. These findings indicate that there is widespread modulation of signaling pathways upon chronic exposure to cigarette smoke in lung cancer cells. Representative MS and MS/MS spectra of hyperphosphorylated phosphopeptides from PAK6 (S560) and TAOK3 (S324) are depicted in Figure 3A and 3B; MS and MS/MS spectra for MAPK14 (Y182) and RPS6KA4 (S347) are depicted in Supplementary Figure S1A and S1B. We also performed motif analysis using motif-X algorithm to identify enriched motifs among the hyperphosphorylated phosphopeptides from H358-S cells [30]. Enriched motifs included, consensus AKT motif (RxxS) (Supplementary Figure S1C). In agreement with the motif analysis, Western blot analysis revealed activation of AKT in H358-S cells compared to H358-P cells (Figure 3C; lanes 1 and 2; pAKT panel).

### AKT mediates PAK6 phosphorylation in NSCLC exposed to cigarette smoke

Several studies have reported overexpression of PAK6 in multiple cancers; however its role in lung cancer is poorly understood. In contrast to group 1 PAK members, PAK6 kinase activity is not stimulated by CDC42 or RAC binding, and therefore the mechanisms that regulate its kinase activity have also not been studied well. Among the members of group II family of PAK kinases, PAK4 has been shown to be upstream regulator of AKT [31, 32]. However, there are no reports that have investigated the crosstalk between PAK6 and AKT signaling. Here, we explored the potential role of AKT in regulating PAK6 activity. Akin to the mass spectrometry data, we observed increased phosphorylation of PAK6 in H358-S cells (Figure 3C - lanes 1 and 2; pPAK6 panel/band). To understand if PAK6 mediated signaling in cigarette smoke treated lung cancer cells is AKT dependent or independent, we treated the H358-S cells with AKT inhibitor (LY294002) and group II PAK inhibitor (PF-3758309). Inhibition of AKT led to decreased AKT phosphorylation at S473 in H358-S cells (Figure 3C –lanes 2 and 4; pAKT panel). We also observed that inhibition of AKT led to decreased phosphorylation of PAK6 in H358-S cells (Figure 3C –lanes 2 and 4; pPAK6 panel/band). Alternatively, inhibition of PAK6 using PF-3758309, did not affect the phosphorylation of AKT in the smoke exposed cells (Figure 3C –lanes 2 and 6; pAKT panel). These results indicate that though PAK6 belongs to the group II family of PAK kinases, unlike PAK4, PAK6 mediated signaling in smoke treated lung cancer cells is activated by AKT.

### Inhibition of PAK6 decreases cellular proliferation in NSCLC cells exposed to cigarette smoke

**Figure 4:**
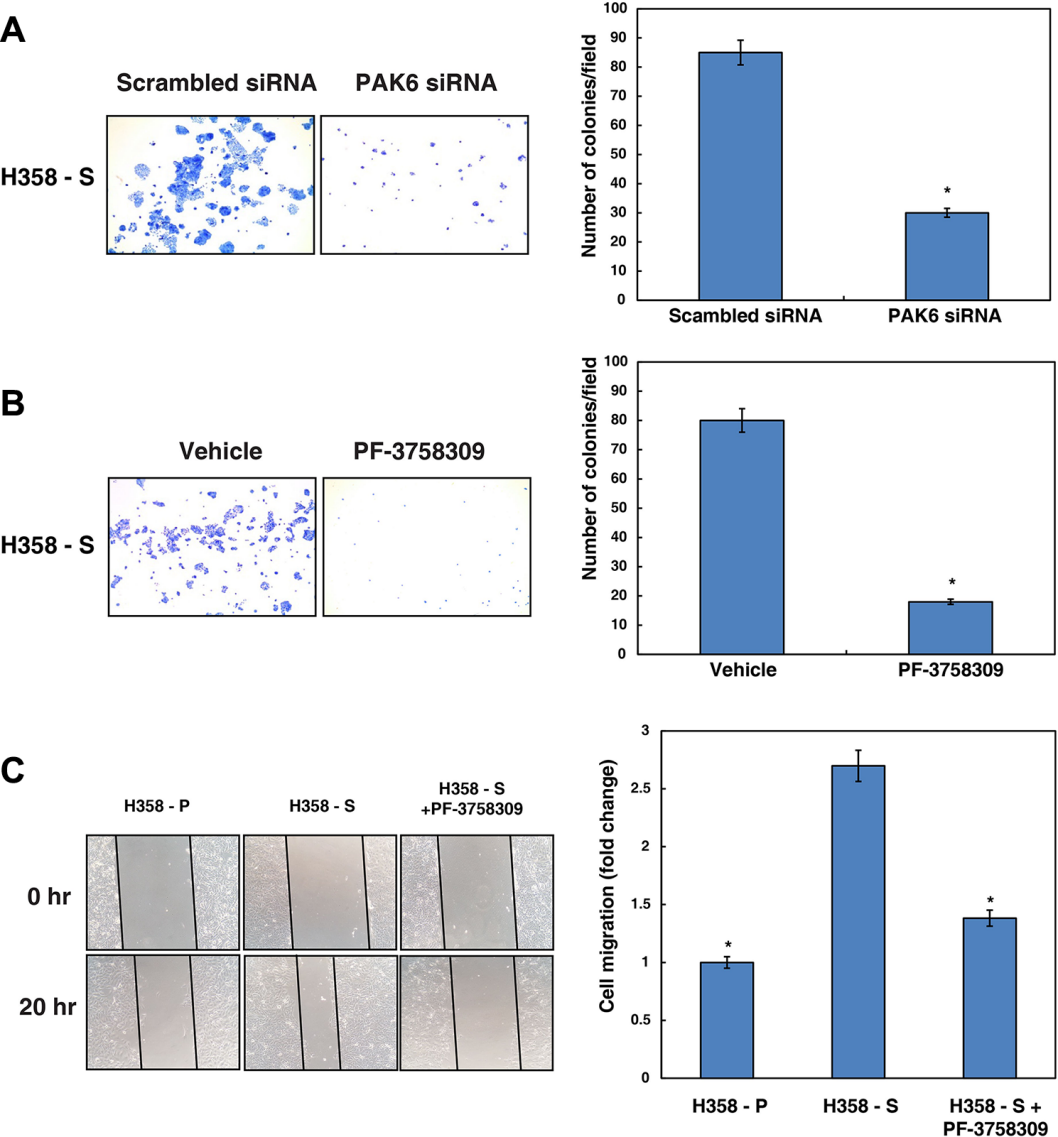
Inhibition of PAK6 decreases cellular proliferation and migratory property of NSCLC cells exposed to cigarette smoke Colony forming assay following (**A**) siRNA knockdown of PAK6 (PAK6 siRNA) or control siRNA (scrambled siRNA) (**B**) inhibition of PAK6 using its inhibitor PF-3758309 or control (vehicle) in H358-S cells. Number of colonies were counted under microscope and represented as bar graph. *p < 0.05. (**C**) Wound migration assays were carried out using H358-P and H358-S cells with or without PF-3758309. Representative photographs are shown from 0 and 20 hrs. Distance migrated by cells was calculated and represented as bar graph. *p < 0.05.

Having observed that PAK6 is activated in H358-S cells and that its signaling is modulated by AKT, we next studied the functional significance of PAK6 in lung cancer. To determine whether PAK6 activity had any effect on cell proliferation, we knocked down the expression of PAK6 in H358-S cells using specific siRNA. Western blot analysis post-transfection with PAK6 siRNA revealed a successful knockdown of PAK6 in H358-S and NSCLC cell lines used in our study (Supplementary Figure S2A). We observed that silencing of PAK6 significantly reduced the colony forming ability of H358-S cells (Figure 4A). In addition, we used an alternative strategy to suppress PAK6 activity using PAK inhibitor (PF-3758309) and examined its effect on proliferation of H358-S and H1299 cells (Supplementary Figure S2B and S2C). Our data clearly shows that proliferation of H358-S and H1299 cells were significantly reduced in the presence of PF-3758309. In agreement with our siRNA data, inhibition of PAK6 using PF-3758309, led to decrease in the colony forming ability of H358-S cells (Figure 4B). Western blot analysis revealed a decrease in p-PAK6 levels in NSCLC cell lines treated with PAK inhibitor (PF-3758309) (Supplementary Figure S2D).

### Inhibition of PAK6 decreases cell motility in NSCLC cells exposed to cigarette smoke

We next studied whether PAK6 has any role in tumor cell motility. To study this, scratch wound assays were carried out using H358-P and H358-S cells with or without PF-3758309. Wounds were made in uniform size and H358-S cells were treated with PF-3758309. After incubation for 20 hours, H358-S cells showed increased migration compared to parental cells. The migratory capacity of H358-S cells was found to be decreased upon PAK6 inhibition. The wound photomicrographs were taken at 0 and 20 hours and distance covered by the cells was measured using Image J software. Images of the wounds at 0 and 20 hours are shown and fold changes in cell migration is depicted in the form of bar graph (Figure 4C). We observed a similar decrease in H1299 cell migration upon treatment with PF-3758039 (Supplementary Figure S2E and S2F). These results indicate that PAK6 plays an essential role in lung cancer cell migration.

### Inhibition of PAK6 decreases the invasive property of NSCLC cells

**Figure 5:**
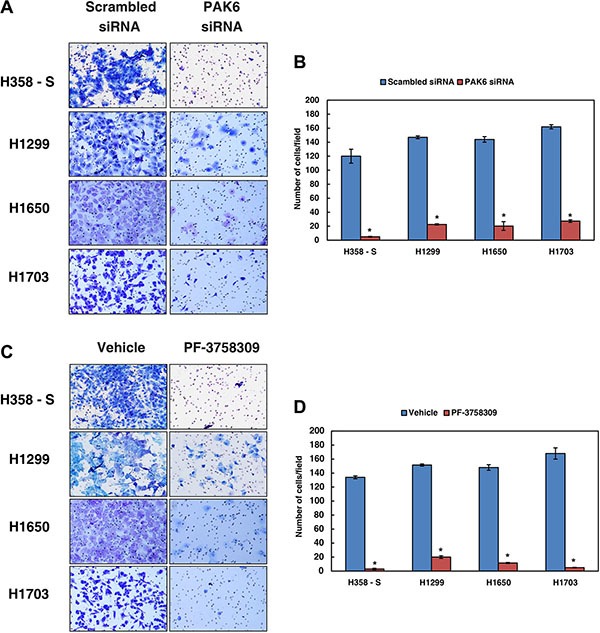
Inhibition of PAK6 decreases the invasive property of NSCLC cells Invasion assays were carried out in a transwell system using Matrigel-coated filters and the number of cells that migrated to the lower chamber was counted. Cells that migrated are visualized following methylene blue staining in H358-S and NSCLC cell lines, H1299, H1650 and H1703, as indicated. (**A**) Cells were transfected with either control (Scrambled) or PAK6 siRNA and invaded cells were photographed (**B**) A graphical representation of the invasive ability of the H358-S and NSCLC cells upon PAK6 silencing *p < 0.05. (**C**) The lung cancer cells were treated with PAK inhibitor PF-3758309 or vehicle (control) and invaded cells were photographed. (**D**) A graphical representation of the invasive ability of the lung cancer cells upon PAK6 inhibition *p < 0.05.

Since inhibition of PAK6 led to a decrease in the migration of lung cancer cells chronically exposed to cigarette smoke, we next studied whether PAK6 has a potential role in regulating invasive potential in H358-S cells and in a panel of NSCLC cell lines established from smokers (H1299, H1650 and H1703). Endogenous expression of PAK6 was knocked down using siRNA or its activity was inhibited using PF-3758309 and invasion assays were performed. Depletion of PAK6 using siRNA resulted in a significant decrease in the invasive ability of the H538-S cells and NSCLC cell lines (Figure 5A and 5B). A similar decrease in the invasive property of cells was observed when PAK6 was inhibited using PF-3758309 in both H358-S and panel of NSCLC cells (Figure 5C–5D). These results suggest that inhibition/silencing of PAK6 can remarkably decrease the metastatic potential of NSCLC.

### Inhibition of PAK6 suppresses tumor growth *in vivo*

**Figure 6:**
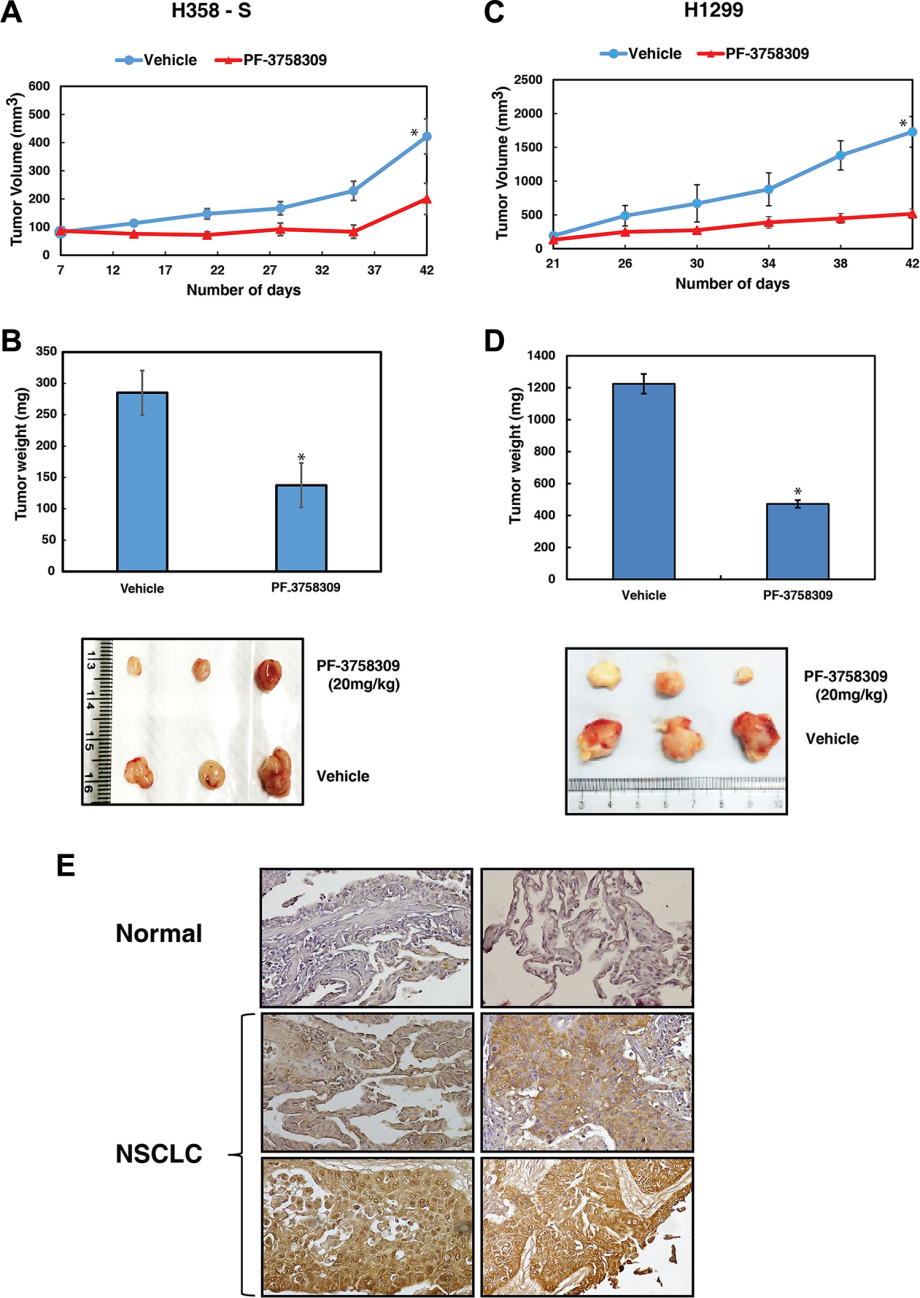
Inhibition of PAK6 suppresses tumor growth *in vivo* (**A**) H358-S (2 × 10^6^) cells were injected into the flanks of NOD-SCID mice (*n* = 5) and tumor growth kinetics is shown as graph. (**B**) Representative pictures and bar graph representing the tumor weights are shown. (**C**) H1299 (2 × 10^6^) cells were injected into the flanks of NOD-SCID mice (*n* = 5) and tumor growth kinetics is represented for a period of 42 days *p < 0.05. (**D**) Bar graph representing the tumor weights *p < 0.05 and representative pictures of tumors from vehicle (DMSO) and PF-3758309 treated groups. (**E**) Immunohistochemical validation of PAK6 in NSCLC cases - representative sections from two NSCLC cases and normal lung tissue stained with anti-PAK6 antibody.

To further corroborate our studies which demonstrate the role of PAK6 in regulating cellular proliferation and invasive potential *in vitro*, we next studied the effect of PAK6 inhibition *in vivo*. H358-S and H1299 (2 × 10^6^) cells were injected subcutaneously (s.c.) into the flanks of NOD-SCID mice. At day 7 and 21, when the tumors reached the size of approximately 50 mm^3^ for H358-S and H1299 respectively, mice were randomized into two groups of five animals each and treated with either vehicle alone (DMSO) or PF-3758309 (20 mg/kg/injection, every 3 days for 3 weeks) intraperitoneally (i.p.). Tumor size was measured every 3 days and the mean tumor volume was calculated. A significant difference (*p* < 0.05) in tumor growth was observed between control and treated group over a 42-day experimental period (Figure 6A and 6C). The mice were sacrificed at the end of 42 days and tumors extracted from PF-3758309 treated group had significant lower tumor mass compared to the vehicle control (Figure 6B and 6D).

### PAK6 is overexpressed in NSCLC

Our results indicate that PAK6 is activated in lung cancer cells in response to cigarette smoke and targeting PAK6 leads to a decrease in oncogenic potential of NSCLC cells. With this we propose that PAK6 can act as a therapeutic target for NSCLC especially in smokers. PAK6 is known to be upregulated in hepatocellular carcinoma and prostate cancer [20, 21]. However, there is no report on the expression of PAK6 in lung cancer. We next studied the expression of PAK6 in primary lung tissue (adenocarcinoma and squamous cell carcinoma) using immunohistochemical staining. Tissue microarray-based immunohistochemical validation was carried out using 78 cases of NSCLC. Staining intensity was scored as negative (0), moderate (1+) or strong (2+). About 66.6 % of the NSCLC showed moderate to strong staining and 94% (48/51) of the normal lung cores showed negative staining. A Chi-square test confirmed that the overexpression of PAK6 in lung tumor tissues was statistically significant (*p-*value = 7.666E^−11^). Representative staining patterns for PAK6 in NSCLC and normal lung tissue are provided in Figure 6E. The results of the immunohistochemical validation are provided in Table 2.

**Table 2:** Summary of the immunohistochemical validation for PAK6 in NSCLC and normal lung tissues

Staining Intensity	Tumor cases	Normal cases
Strong	18	1
Moderate	34	2
Negative	26	48
*p*-value of significant difference between tumor and normal groups (Chi-square test)	7.66E^−11^	

The table lists staining intensities for tumor and normal cases and *p*-value.

## DISCUSSION

Cigarette smoking remains the leading cause for lung cancer and recent studies have shown distinct molecular signatures in lung tumors based on smoking habits, suggesting the existence of divergent mechanisms of tumorigenesis in smokers and non-smokers [33–35] However, molecular signaling induced in response to cigarette smoke remains incompletely characterized. Here, using a cell line model we have attempted to delineate the altered signaling in response to chronic cigarette smoke exposure. Studies which have investigated the effect of CSC have reported epithelial to mesenchymal transition, increased cell migration and decreased apoptosis in lung cell lines treated with CSC [36, 37]. Consistent with previously published studies, H358-S cells showed increased migration, invasion and proliferative capacities when compared to the parental cells. Some of the signaling pathways reported to be induced in response to cigarette smoke include phosphatidylinositol 3-kinase (PI3K)/AKT, Ras/mitogen-activated protein kinases (MAPKs) and NF-κΒ [38, 39]. It has been shown that activation of NF-κΒ in response to cigarette smoke upregulates BCL-XL leading to survival in human bronchial epithelial cells [39]. In concordance with these studies, we observed an increased expression of NF-κΒ and BCL-XL in H358 cells chronically treated with cigarette smoke.

There are limited reports on proteomic alterations in lung cancer upon acute exposure to CSC. Such studies have identified increased expression of receptor for advanced glycation endpoints (RAGE), thioredoxin (Trx) and upregulation of kinases like ERK1/2, MEK6 and RSK1 [40–43]. However, there are no phosphoproteomic studies investigating the global alteration in signaling pathways upon chronic smoke exposure. Our data demonstrates widespread alterations in signaling mechanism(s) in H358-S cells. We observed hyperphosphorylation of the activation sites of several kinases and their downstream effectors in cigarette smoke exposed cells. We have identified significant phosphorylation of STAT3 (S726) (4.6 - fold) in response to cigarette smoke in our cell line model. The findings are concordant with recent report which has shown that cigarette smoke induces MMP2 and MMP9 expressions through activation of JAK2/STAT3 pathway [44]. It is established that overexpression of AKT plays a key role in tumorigenesis and cancer progression [45]. Aberrant activation of phosphatidylinositol 3-kinase (PI3K) signaling pathway has been identified in a wide range of cancers [46, 47]. PI3K/AKT/mTOR pathway is under investigation as it is activated by multiple signaling nodes such as EGFR, IGF-1R, c-MET. Currently, number of PI3K/mTOR inhibitors such as RAD001, BEZ235 and XL765 are under investigation either in combination or with other EGFR TKIs [48–50]. However, their efficacy still needs to be tested in a large cohort of NSCLC patients. We report an enrichment of consensus AKT motif and activation of AKT (increased phosphorylation at S473) among the hyperphosphorylated phosphopeptides in the smoke treated cells, which is suggestive of active PI3K/AKT signaling in these cells. The other kinases identified in our study included TAOK3 (S324), MAPK14 (Y182) and PAK6 (S560) amongst others. TAOKs are Ste20p-related MAP kinases (MAP3Ks) that activate p38 MAPK in response to genotoxic insults [29]. Chronic exposure to cigarette smoke is known to be genotoxic and induces genomic alterations [51, 52]. TAOKs are reported to mediate ATM/ATR induced activation of p38 upon DNA damage. p38 MAPK is known to be a stress sensor which regulates cell cycle check points and activates PAK6 [28]. PAK isoforms are known to influence multiple cellular processes including cell proliferation, invasion and migration [27, 53].

PAK6 belongs to group II PAKs family, contains a kinase domain and N-terminal CRIB domain but lacks N-terminal auto inhibitory domain. Genomic amplification of PAK4 is reported in ovarian and pancreatic cancers [54, 55]. PAK4 overexpression is reported in various cancers including ovarian, colon and gastric cancers [56–58]. PAK5 is reported to be overexpressed in colorectal and gastric cancer [59, 60]. Liu *et al* have recently reported overexpression of group I PAK in NSCLC tissues [61]. Another study independently have shown overexpression of PAK4 and increased expression correlated with poor outcome in NSCLC [26]. In our study, we have identified PAK6 to be significantly phosphorylated at S560. S560 is located in the activation loop of PAK6 and is the autophosphorylation site of PAK6, and its mutation leads to blockade of PAK6 activation by MKK6 [28]. Unlike other members of PAK family; limited information is available on role of PAK6 in tumorigenesis. PAK6 is known to be overexpressed in hepatocellular carcinoma and prostate cancers [20, 21]. PAK6 is also known to bind ER-alpha and this binding is known to increase upon administration of tamoxifen [62]. In prostate cancer, chemo-sensitivity to docetaxel was enhanced when used in combination with PAK6 siRNA [63]. PAK6 has also been linked to radiosensitivity of prostate cancer cells. PAK6 inhibition, in combination with irradiation results in significant decrease in prostate cancer cell survival [64].

Our findings indicate that PAK6 plays a crucial role in both proliferation and metastatic potential of lung cancer cells in response to cigarette smoke. We demonstrate here that chronic exposure to cigarette smoke leads to activation of AKT and in turn PAK6. Consistent with our *in vitro* assays, inhibition of PAK6 *in vivo* also showed a reduction in lung tumor growth. In this study, tissue microarray-based immunohistochemical staining revealed overexpression of PAK6 in more than 66.6% of the NSCLC cases, which again corroborates with our *in vitro* findings. These results indicate PAK6 as a novel potential target for NSCLC, especially in smokers. Our findings further highlight the need for systematic investigation of PAK6 as a potential therapeutic target for lung cancer in a larger cohort of patients.

## MATERIALS AND METHODS

### Cell culture and SILAC labeling

Human lung cancer cell line H358, H1650, H1299 and H1703 were obtained from American Type Culture Collection (ATCC, Manassas, VA). H358 were grown in DMEM and H1650, H1299 and H1703 were maintained in RPMI containing 10% fetal bovine serum (Clontech, Mountain View, CA) and 1% penicillin/streptomycin mixture at 37°C in a humidified 5% CO_2_ atmosphere. To study the effect of the cigarette smoke condensate (CSC, Murty Pharmaceuticals, Inc., KY), H358 cells were grown in the smoking dedicated incubator [22]. H358 cells were subjected to chronic treatment with 0.1% CSC for 12 months [65]. Cells that were grown in a normal incubator that did not have any cell lines treated with CSC are labeled as control or parental (H358-P). The H358-P cells were then adapted to SILAC media enriched with ^13^C_6_-lysine and ^13^C_6_-arginine and H358-S cells were maintained in regular media [66].

### Trypsin digestion and basic pH reverse phase liquid chromatography

Post twelve hours of serum starvation, cells were harvested and lysed in urea lysis buffer (20 mM HEPES pH 8.0, 9 M urea, 1 mM sodium orthovanadate, 2.5 mM sodium pyrophosphate, 1 mM phosphoglycerophosphate). Protein concentration was determined using BCA assays. Five mg of lysate from treated and untreated conditions were mixed. The mixture was reduced with dithiothreitol at 60°C and alkylated using iodoacetamide at room temperature. Concentration of urea was brought to 2 M using HEPES buffer and digested overnight at 37°C using modified trypsin (Promega). The protein digests were loaded onto Sep-Pak C^18^ column which was washed with TFA and eluted using 40% ACN with 0.1% TFA. The peptide samples were lyophilized and subjected to basic pH reverse phase chromatography (bRPLC). The lyophilized samples were reconstituted in bRPLC solvent A (7 mM TAEBC, pH 9) and were separated on XBridge BEH C_18_ Column (Waters, UK) with a linear increase in gradient from 5 to 100% of 7 mM TEABC with 90% acetonitrile (pH 9) over 30 min. and persisting for 10 minutes. For each condition, 24 fractions were collected and dried before LC-MS/MS analysis.

### Titanium dioxide-based phosphopeptide enrichment

The peptides from each fraction were further enriched for phosphopeptides using TiO_2-_based enrichment method [67]. Briefly, the TiO_2_ beads were washed in 5% 2, 5-dihydroxybenzoic acid (DHB) for 2 hours on the rotator at room temperature. The bRPLC peptide fractions were dissolved in 5% DHB and incubated with TiO_2_ beads for 30 minutes on a rotator at room temperature. The phosphopeptide-TiO_2_ beads were washed several times and eluted thrice with 2% ammonia. The enriched peptides were concentrated by vacuum centrifugation and desalted using C_18_ StageTips. The enriched and desalted peptide samples were further subjected to mass spectrometry analysis.

### LC-MS/MS and data analysis

LC-MS/MS analysis of enriched phosphopeptides was carried out using Eksigent nanoflow liquid chromatography interfaced with a LTQ-Orbitrap Velos mass spectrometer (Thermo Scientific, San Jose, CA). The peptides were loaded onto a 2 cm × 75 μm, Magic C_18_ AQ 5 μm, 120 Å trap column at a flow rate of 3 μl/min using 0.1% formic acid for enrichment. The peptides were separated on an analytical column (10 cm × 75 μm, Magic C_18_ AQ 5 μm, 120 Å) by a linear gradient from 5 to 60% ACN in 90 minutes. MS and MS/MS scans were acquired at resolving power of 60,000 and 15.000 at 400 m/z, respectively. HCD fragmentation of the 10 most abundant ions was carried out in a data dependent manner (isolation width: 1.90 m/z; normalized collision energy: 35%). The tandem mass spectrometry data were searched using MASCOT (v 2.2) and SEQUEST search algorithms against a Human RefSeq database (RefSeq 59) supplemented with frequently observed contaminants through the Proteome Discoverer platform (v1.3, Thermo Scientific, Bremen, Germany). For both algorithms, the search parameters included a maximum of 2 missed cleavage; carbamidomethylation at cysteine as a fixed modification, oxidation at methionine, phosphorylation at serine, threonine and tyrosine and SILAC labels ^13^C_6_-Lysine; ^13^C_6_-Arginine as variable modifications. The MS error tolerance was set at 20 ppm and MS/MS error tolerance to 0.1 Da. The data were searched against a decoy database and the results from both searches were used to estimate *q* values using the Percolator algorithm within the Proteome Discoverer suite. Peptides were considered identified at a *q* value of <0.01 [68]. The probability of phosphorylation for each S/T/Y site on each peptide was calculated by the PhosphoRS node (Version 3.0) in the Proteome Discoverer [69]. Peptides with ≥ 75% phosphosites probability were considered for further analysis. Identification of enriched motifs was carried out using motif-X algorithm [30]. Phosphowindow of 15 aa long was used for extracting consensus motif.

### LC-MS/MS data availability

The raw data has been submitted to ProteomeXchange Consortium (http://www.proteomecentral.proteomexchange.org) via the PRIDE public data repository [70] and can be accessed using the data identifier – PXD003108.

### Western blotting

Whole cell extracts of H358-P and H358-S cells were prepared using modified RIPA lysis buffer (Merck Millipore, Billerica, MA,) containing protease inhibitors (Roche, Indianapolis, IN,) and phosphatase inhibitors (Thermo Scientific, Bremen, Germany). Western blot analysis was performed as previously described using 30 μg protein lysates [65, 71]. Nitrocellulose membranes were hybridized with primary antibodies and developed using Luminol reagent (Santa Cruz Biotechnology, Dallas, TX,) as per the manufacturer's instructions. Anti-PAK6 antibody was obtained from Novus (Novus Biologicals, Littleton CO), Phospho-PAK6 (Ser 560), Anti-AKT and phospho-AKT (Ser 473) antibodies were obtained from Cell Signaling Technology (Cell Signaling Technology, Beverly, MA). Beta-actin antibody was obtained from Sigma (St. Louis, MO).

### Cell proliferation assays

Cell proliferation assays were carried out as described previously [72]. H358-P and H358-S cells were seeded at a density of 12,000 cells/well into 6-well plate and cells were counted subsequently after every 48 hours. Cell proliferation was determined over a period of 10 days and growth kinetics was plotted [73]. All experiments were carried out in triplicates.

### siRNA transfection

ON-TARGETplus SMARTpool control siRNA and PAK6 siRNA were obtained from Dharmacon (Lafayette, CO). The H358-smoke, H1299, H1650 and H1703 cells were transfected with RNAiMAX (Invitrogen, Grand Island, NY) following manufacturer's instructions. Transfection was carried out as previously described [65, 71]. Cells were subjected to invasion assays and colony formation assays 48 hours post-transfection, unless otherwise stated.

### Colony formation assays

H358-S cells were treated with either PAK6 inhibitor PF-3758309 or transfected with PAK6 siRNA or scramble siRNA. 48 h post transfection, 3 × 10^3^ cells were seeded in 6-well plates with complete media. The resulting colonies were fixed with methanol, and stained with Giemsa (Sigma, St. Louis, MO). The number of colonies per dish was counted. All experiments were performed in triplicate and standard deviation was calculated.

### Wound migration assays

The wound migration assays were performed as described previously [74]. Briefly, the cells were seeded and uniform size wound was introduced in each condition. H358- S and H1299 were treated with PAK6 inhibitor PF-3758309 or vehicle control. The cells were allowed to grow for 20 hours. The wound photomicrographs at 0 and 20 hours were taken under microscope. All experiments were performed in triplicate unless otherwise indicated.

### Invasion assays

Invasion assays were performed in a transwell system (BD Biosciences, San Jose, CA) with Matrigel-coated filters, and cellular invasion was evaluated after 48 hours as described [71, 75]. Briefly, invasive property of the cells was assayed in the membrane invasion culture system using PET membrane (8-μm pore size). The cells were seeded at density of 2.0 × 10^4^ cells in 500 μl of serum free media on the Matrigel-coated PET membrane in the upper compartment. The lower compartment was filled with complete growth media and the plates were incubated at 37°C for 48 hours. Post incubation time, the upper surface of the membrane was wiped with a cotton-tip applicator to remove non-migratory cells. Cells that migrated to the lower surface of membrane were fixed and stained by methylene blue. Each measurement was performed in duplicate and the experiments were repeated three times.

### *In vivo* studies

Xenografts were generated in 6-week-old NOD-SCID male mice with H358-S and H1299 (2 × 10^6^) cells. Ten mice with successfully engrafted H358-S and H1299 xenografts were randomized into two cohorts of five animals per group and were treated with either DMSO or PF-3758309 (20 mg/kg/injection, every 3 days) intraperitoneally (i.p.). Tumor size was measured after every 3 days using digital calipers (Fisher Scientific) upto three weeks and the mean tumor volume was calculated using the formula (π/6(d1xd2)^3/2^) [76]. Mice were housed in pathogen free conditions at the experimental animal facility of Indian Institute of Sciences, Bangalore, India and the experiments described here were conducted according to the institutional ethical guidelines.

### Immunohistochemistry

A semi-quantitative assessment was performed to evaluate the immunoreactivity as described previously [77]. Briefly, the formalin fixed paraffin embedded tissue sections were deparaffinised and antigen retrieval was carried out using heat-induced epitope retrieval by incubating them for 20 minutes in antigen retrieval buffer (0.01 M Trisodium citrate buffer, pH 6). The quenching of endogenous peroxidases was done by using a blocking solution followed by washes with wash buffer (PBS with 0.05% Tween-20). The sections were incubated with primary antibody overnight at 4°C in a humidified chamber. Anti-PAK6 (Novus Biologicals, Littleton CO) was used at 1:1500 dilution. After incubation with the primary antibody, the sections were washed thrice with wash buffer. The slides were incubated with appropriate horseradish peroxidase conjugated rabbit secondary antibody for 30 minutes at room temperature. Excess secondary antibody was removed using wash buffer followed by addition of DAB substrate. The signal was developed using DAB chromogen (DAKO, Glostrup, Denmark). Tissue sections were then observed under the microscope. The immunohistochemical labeling was assessed by an experienced pathologist. The intensity of staining was scored on a grading scale ranging from 0 to 2+, where 0 represented negative staining, 1+ represented moderate staining, and 2+ represented strong staining. To determine statistical significance of difference of expression chi-square test was carried out.

### Statistical analysis

Statistical analysis was performed using an open source program R. The data were considered statistically significant if *p* < 0.05. Wound assay and *in vitro* tube formation data were quantified using the Image Pro plus 6.0 software (Media Cybernetics, Rockville, MD, USA).

## SUPPLEMENTARY MATERIALS FIGURES AND TABLES





## References

[1] Lozano R, Naghavi M, Foreman K, Lim S, Shibuya K, Aboyans V, Abraham J, Adair T, Aggarwal R, Ahn SY, Alvarado M, Anderson HR, Anderson LM (2012). Global and regional mortality from 235 causes of death for 20 age groups in 1990 and 2010: a systematic analysis for the Global Burden of Disease Study 2010. Lancet.

[2] Jha P (2009). Avoidable global cancer deaths and total deaths from smoking. Nat Rev Cancer.

[3] Herbst RS, Heymach JV, Lippman SM (2008). Lung cancer. N Engl J Med.

[4] Molina JR, Yang P, Cassivi SD, Schild SE, Adjei AA (2008). Non-small cell lung cancer: epidemiology, risk factors, treatment, and survivorship. Mayo Clin Proc.

[5] Warren GW, Cummings KM (2013). Tobacco and lung cancer: risks, trends, and outcomes in patients with cancer. Am Soc Clin Oncol Educ Book.

[6] Govindan R, Ding L, Griffith M, Subramanian J, Dees ND, Kanchi KL, Maher CA, Fulton R, Fulton L, Wallis J, Chen K, Walker J, McDonald S (2012). Genomic landscape of non-small cell lung cancer in smokers and never-smokers. Cell.

[7] Pao W, Miller V, Zakowski M, Doherty J, Politi K, Sarkaria I, Singh B, Heelan R, Rusch V, Fulton L, Mardis E, Kupfer D, Wilson R (2004). EGF receptor gene mutations are common in lung cancers from “never smokers” and are associated with sensitivity of tumors to gefitinib and erlotinib. Proc Natl Acad Sci USA.

[8] Lynch TJ, Bell DW, Sordella R, Gurubhagavatula S, Okimoto RA, Brannigan BW, Harris PL, Haserlat SM, Supko JG, Haluska FG, Louis DN, Christiani DC, Settleman J (2004). Activating mutations in the epidermal growth factor receptor underlying responsiveness of non-small-cell lung cancer to gefitinib. N Engl J Med.

[9] Filosto S, Becker CR, Goldkorn T (2012). Cigarette smoke induces aberrant EGF receptor activation that mediates lung cancer development and resistance to tyrosine kinase inhibitors. Mol Cancer Ther.

[10] Fidler MJ, Shersher DD, Borgia JA, Bonomi P (2012). Targeting the insulin-like growth factor receptor pathway in lung cancer: problems and pitfalls. Ther Adv Med Oncol.

[11] Aoshiba K, Tamaoki J, Nagai A (2001). Acute cigarette smoke exposure induces apoptosis of alveolar macrophages. Am J Physiol Lung Cell Mol Physiol.

[12] Carnevali S, Petruzzelli S, Longoni B, Vanacore R, Barale R, Cipollini M, Scatena F, Paggiaro P, Celi A, Giuntini C (2003). Cigarette smoke extract induces oxidative stress and apoptosis in human lung fibroblasts. Am J Physiol Lung Cell Mol Physiol.

[13] Kode A, Yang SR, Rahman I (2006). Differential effects of cigarette smoke on oxidative stress and proinflammatory cytokine release in primary human airway epithelial cells and in a variety of transformed alveolar epithelial cells. Respir Res.

[14] Ramage L, Jones AC, Whelan CJ (2006). Induction of apoptosis with tobacco smoke and related products in A549 lung epithelial cells *in vitro*. J Inflamm (Lond).

[15] Wu X, Renuse S, Sahasrabuddhe NA, Zahari MS, Chaerkady R, Kim MS, Nirujogi RS, Mohseni M, Kumar P, Raju R, Zhong J, Yang J, Neiswinger J (2014). Activation of diverse signalling pathways by oncogenic PIK3CA mutations. Nat Commun.

[16] Zhong J, Kim MS, Chaerkady R, Wu X, Huang TC, Getnet D, Mitchell CJ, Palapetta SM, Sharma J, O'Meally RN, Cole RN, Yoda A, Moritz A (2012). TSLP signaling network revealed by SILAC-based phosphoproteomics. Mol Cell Proteomics.

[17] Ong SE, Blagoev B, Kratchmarova I, Kristensen DB, Steen H, Pandey A, Mann M (2002). Stable isotope labeling by amino acids in cell culture, SILAC, as a simple and accurate approach to expression proteomics. Mol Cell Proteomics.

[18] Dammann K, Khare V, Gasche C (2014). Tracing PAKs from GI inflammation to cancer. Gut.

[19] Manser E, Leung T, Salihuddin H, Zhao ZS, Lim L (1994). A brain serine/threonine protein kinase activated by Cdc42 and Rac1. Nature.

[20] Chen H, Miao J, Li H, Wang C, Li J, Zhu Y, Wang J, Wu X, Qiao H (2014). Expression and prognostic significance of p21-activated kinase 6 in hepatocellular carcinoma. J Surg Res.

[21] Kaur R, Yuan X, Lu ML, Balk SP (2008). Increased PAK6 expression in prostate cancer and identification of PAK6 associated proteins. Prostate.

[22] Kim MS, Huang Y, Lee J, Zhong X, Jiang WW, Ratovitski EA, Sidransky D (2010). Cellular transformation by cigarette smoke extract involves alteration of glycolysis and mitochondrial function in esophageal epithelial cells. Int J Cancer.

[23] Plati J, Bucur O, Khosravi-Far R (2011). Apoptotic cell signaling in cancer progression and therapy. Integr Biol (Camb).

[24] Kajihara R, Fukushige S, Shioda N, Tanabe K, Fukunaga K, Inui S (2010). CaMKII phosphorylates serine 10 of p27 and confers apoptosis resistance to HeLa cells. Biochem Biophys Res Commun.

[25] Zhou BP, Liao Y, Xia W, Zou Y, Spohn B, Hung MC (2001). HER-2/neu induces p53 ubiquitination via Akt-mediated MDM2 phosphorylation. Nat Cell Biol.

[26] Cai S, Ye Z, Wang X, Pan Y, Weng Y, Lao S, Wei H, Li L (2015). Overexpression of P21-activated kinase 4 is associated with poor prognosis in non-small cell lung cancer and promotes migration and invasion. J Exp Clin Cancer Res.

[27] Ryu BJ, Lee H, Kim SH, Heo JN, Choi SW, Yeon JT, Lee J, Cho JY, Lee SY (2014). PF-3758309, p21-activated kinase 4 inhibitor, suppresses migration and invasion of A549 human lung cancer cells via regulation of CREB, NF-kappaB, and beta-catenin signalings. Mol Cell Biochem.

[28] Kaur R, Liu X, Gjoerup O, Zhang A, Yuan X, Balk SP, Schneider MC, Lu ML (2005). Activation of p21-activated kinase 6 by MAP kinase kinase 6 and p38 MAP kinase. J Biol Chem.

[29] Raman M, Earnest S, Zhang K, Zhao Y, Cobb MH (2007). TAO kinases mediate activation of p38 in response to DNA damage. EMBO J.

[30] Schwartz D, Gygi SP (2005). An iterative statistical approach to the identification of protein phosphorylation motifs from large-scale data sets. Nat Biotechnol.

[31] Fu X, Feng J, Zeng D, Ding Y, Yu C, Yang B (2014). PAK4 confers cisplatin resistance in gastric cancer cells via PI3K/Akt- and MEK/Erk-dependent pathways. Biosci Rep.

[32] Tyagi N, Bhardwaj A, Singh AP, McClellan S, Carter JE, Singh S (2014). p-21 activated kinase 4 promotes proliferation and survival of pancreatic cancer cells through AKT- and ERK-dependent activation of NF-kappaB pathway. Oncotarget.

[33] Hu Y, Chen G (2015). Pathogenic mechanisms of lung adenocarcinoma in smokers and non-smokers determined by gene expression interrogation. Oncol Lett.

[34] Staaf J, Jonsson G, Jonsson M, Karlsson A, Isaksson S, Salomonsson A, Pettersson HM, Soller M, Ewers SB, Johansson L, Jonsson P, Planck M (2012). Relation between smoking history and gene expression profiles in lung adenocarcinomas. BMC Med Genomics.

[35] Vucic EA, Thu KL, Pikor LA, Enfield KS, Yee J, English JC, MacAulay CE, Lam S, Jurisica I, Lam WL (2014). Smoking status impacts microRNA mediated prognosis and lung adenocarcinoma biology. BMC Cancer.

[36] Nagathihalli NS, Massion PP, Gonzalez AL, Lu P, Datta PK (2012). Smoking induces epithelial-to-mesenchymal transition in non-small cell lung cancer through HDAC-mediated downregulation of E-cadherin. Mol Cancer Ther.

[37] Samanta D, Kaufman J, Carbone DP, Datta PK (2012). Long-term smoking mediated down-regulation of Smad3 induces resistance to carboplatin in non-small cell lung cancer. Neoplasia.

[38] Ibuki Y, Toyooka T, Zhao X, Yoshida I (2014). Cigarette sidestream smoke induces histone H3 phosphorylation via JNK and PI3K/Akt pathways, leading to the expression of proto-oncogenes. Carcinogenesis.

[39] Liu X, Togo S, Al-Mugotir M, Kim H, Fang Q, Kobayashi T, Wang X, Mao L, Bitterman P, Rennard S (2008). NF-kappaB mediates the survival of human bronchial epithelial cells exposed to cigarette smoke extract. Respir Res.

[40] Bortner JD, Richie JP, Das A, Liao J, Umstead TM, Stanley A, Stanley BA, Belani CP, El-Bayoumy K (2011). Proteomic profiling of human plasma by iTRAQ reveals down-regulation of ITI-HC3 and VDBP by cigarette smoking. J Proteome Res.

[41] Carter CA, Misra M, Pelech S (2011). Proteomic analyses of lung lysates from short-term exposure of Fischer 344 rats to cigarette smoke. J Proteome Res.

[42] Sexton K, Balharry D, Brennan P, McLaren J, Brewis IA, BeruBe KA (2011). Proteomic profiling of human respiratory epithelia by iTRAQ reveals biomarkers of exposure and harm by tobacco smoke components. Biomarkers.

[43] Zhang S, Xu N, Nie J, Dong L, Li J, Tong J (2008). Proteomic alteration in lung tissue of rats exposed to cigarette smoke. Toxicol Lett.

[44] Ghosh A, Pechota A, Coleman D, Upchurch GR, Eliason JL (2015). Cigarette smoke-induced MMP2 and MMP9 secretion from aortic vascular smooth cells is mediated via the Jak/Stat pathway. Hum Pathol.

[45] Oka N, Tanimoto S, Taue R, Nakatsuji H, Kishimoto T, Izaki H, Fukumori T, Takahashi M, Nishitani M, Kanayama HO (2006). Role of phosphatidylinositol-3 kinase/Akt pathway in bladder cancer cell apoptosis induced by tumor necrosis factor-related apoptosis-inducing ligand. Cancer Sci.

[46] Engelman JA, Luo J, Cantley LC (2006). The evolution of phosphatidylinositol 3-kinases as regulators of growth and metabolism. Nat Rev Genet.

[47] Samuels Y, Wang Z, Bardelli A, Silliman N, Ptak J, Szabo S, Yan H, Gazdar A, Powell SM, Riggins GJ, Willson JK, Markowitz S, Kinzler KW (2004). High frequency of mutations of the PIK3CA gene in human cancers. Science.

[48] http://clinicaltrials.gov/show/NCT00777699.

[49] Papadopoulos KP, Tabernero J, Markman B, Patnaik A, Tolcher AW, Baselga J, Shi W, Egile C, Ruiz-Soto R, Laird AD, Miles D, Lorusso PM (2014). Phase I safety, pharmacokinetic, and pharmacodynamic study of SAR245409 (XL765), a novel, orally administered PI3K/mTOR inhibitor in patients with advanced solid tumors. Clin Cancer Res.

[50] Xu CX, Li Y, Yue P, Owonikoko TK, Ramalingam SS, Khuri FR, Sun SY (2011). The combination of RAD001 and NVP-BEZ235 exerts synergistic anticancer activity against non-small cell lung cancer *in vitro* and *in vivo*. PLoS One.

[51] DeMarini DM (2004). Genotoxicity of tobacco smoke and tobacco smoke condensate: a review. Mutat Res.

[52] Huang YT, Lin X, Liu Y, Chirieac LR, McGovern R, Wain J, Heist R, Skaug V, Zienolddiny S, Haugen A, Su L, Fox EA, Wong KK (2011). Cigarette smoking increases copy number alterations in nonsmall-cell lung cancer. Proc Natl Acad Sci USA.

[53] Minden A (2012). PAK4–6 in cancer and neuronal development. Cell Logist.

[54] Chen S, Auletta T, Dovirak O, Hutter C, Kuntz K, El-ftesi S, Kendall J, Han H, Von Hoff DD, Ashfaq R, Maitra A, Iacobuzio-Donahue CA, Hruban RH (2008). Copy number alterations in pancreatic cancer identify recurrent PAK4 amplification. Cancer Biol Ther.

[55] Davis SJ, Sheppard KE, Pearson RB, Campbell IG, Gorringe KL, Simpson KJ (2013). Functional analysis of genes in regions commonly amplified in high-grade serous and endometrioid ovarian cancer. Clin Cancer Res.

[56] Ahn HK, Jang J, Lee J, Se Hoon P, Park JO, Park YS, Lim HY, Kim KM, Kang WK (2011). P21-activated kinase 4 overexpression in metastatic gastric cancer patients. Transl Oncol.

[57] Callow MG, Clairvoyant F, Zhu S, Schryver B, Whyte DB, Bischoff JR, Jallal B, Smeal T (2002). Requirement for PAK4 in the anchorage-independent growth of human cancer cell lines. J Biol Chem.

[58] Siu MK, Chan HY, Kong DS, Wong ES, Wong OG, Ngan HY, Tam KF, Zhang H, Li Z, Chan QK, Tsao SW, Stromblad S, Cheung AN (2010). p21-activated kinase 4 regulates ovarian cancer cell proliferation, migration, and invasion and contributes to poor prognosis in patients. Proc Natl Acad Sci USA.

[59] Gong W, An Z, Wang Y, Pan X, Fang W, Jiang B, Zhang H (2009). P21-activated kinase 5 is overexpressed during colorectal cancer progression and regulates colorectal carcinoma cell adhesion and migration. Int J Cancer.

[60] Gu J, Li K, Li M, Wu X, Zhang L, Ding Q, Wu W, Yang J, Mu J, Wen H, Lu J, Hao Y, Chen L (2013). A role for p21-activated kinase 7 in the development of gastric cancer. FEBS J.

[61] Liu Y, Wang S, Dong QZ, Jiang GY, Han Y, Wang L, Wang EH (2016). The P21-activated kinase expression pattern is different in non-small cell lung cancer and affects lung cancer cell sensitivity to epidermal growth factor receptor tyrosine kinase inhibitors. Med Oncol.

[62] Lee SR, Ramos SM, Ko A, Masiello D, Swanson KD, Lu ML, Balk SP (2002). AR and ER interaction with a p21-activated kinase (PAK6). Mol Endocrinol.

[63] Wen X, Li X, Liao B, Liu Y, Wu J, Yuan X, Ouyang B, Sun Q, Gao X (2009). Knockdown of p21-activated kinase 6 inhibits prostate cancer growth and enhances chemosensitivity to docetaxel. Urology.

[64] Zhang M, Siedow M, Saia G, Chakravarti A (2010). Inhibition of p21-activated kinase 6 (PAK6) increases radiosensitivity of prostate cancer cells. Prostate.

[65] Chang X, Ravi R, Pham V, Bedi A, Chatterjee A, Sidransky D (2011). Adenylate kinase 3 sensitizes cells to cigarette smoke condensate vapor induced cisplatin resistance. PLoS One.

[66] Sahasrabuddhe NA, Huang TC, Ahmad S, Kim MS, Yang Y, Ghosh B, Leach SD, Gowda H, Somani BL, Chaerkady R, Pandey A (2014). Regulation of PPAR-alpha pathway by Dicer revealed through proteomic analysis. J Proteomics.

[67] Thingholm TE, Jorgensen TJ, Jensen ON, Larsen MR (2006). Highly selective enrichment of phosphorylated peptides using titanium dioxide. Nat Protoc.

[68] Kall L, Canterbury JD, Weston J, Noble WS, MacCoss MJ (2007). Semi-supervised learning for peptide identification from shotgun proteomics datasets. Nat Methods.

[69] Taus T, Kocher T, Pichler P, Paschke C, Schmidt A, Henrich C, Mechtler K (2011). Universal and confident phosphorylation site localization using phosphoRS. J Proteome Res.

[70] Vizcaino JA, Cote RG, Csordas A, Dianes JA, Fabregat A, Foster JM, Griss J, Alpi E, Birim M, Contell J, O'Kelly G, Schoenegger A, Ovelleiro D (2013). The PRoteomics IDEntifications (PRIDE) database and associated tools: status in 2013. Nucleic Acids Res.

[71] Subbannayya Y, Syed N, Barbhuiya MA, Raja R, Marimuthu A, Sahasrabuddhe N, Pinto SM, Manda SS, Renuse S, Manju HC, Zameer MA, Sharma J, Brait M (2015). Calcium calmodulin dependent kinase kinase 2 - a novel therapeutic target for gastric adenocarcinoma. Cancer Biol Ther.

[72] Syed N, Chavan S, Sahasrabuddhe NA, Renuse S, Sathe G, Nanjappa V, Radhakrishnan A, Raja R, Pinto SM, Srinivasan A, Prasad TS, Srikumar K, Gowda H (2015). Silencing of high-mobility group box 2 (HMGB2) modulates cisplatin and 5-fluorouracil sensitivity in head and neck squamous cell carcinoma. Proteomics.

[73] Chatterjee A, Mambo E, Zhang Y, Deweese T, Sidransky D (2006). Targeting of mutant hogg1 in mammalian mitochondria and nucleus: effect on cellular survival upon oxidative stress. BMC Cancer.

[74] Behera R, Kumar V, Lohite K, Karnik S, Kundu GC (2010). Activation of JAK2/STAT3 signaling by osteopontin promotes tumor growth in human breast cancer cells. Carcinogenesis.

[75] Ebrahimnejad A, Streichert T, Nollau P, Horst AK, Wagener C, Bamberger AM, Brummer J (2004). CEACAM1 enhances invasion and migration of melanocytic and melanoma cells. Am J Pathol.

[76] Raja R, Kale S, Thorat D, Soundararajan G, Lohite K, Mane A, Karnik S, Kundu GC (2014). Hypoxia-driven osteopontin contributes to breast tumor growth through modulation of HIF1alpha-mediated VEGF-dependent angiogenesis. Oncogene.

[77] Kashyap MK, Marimuthu A, Kishore CJ, Peri S, Keerthikumar S, Prasad TS, Mahmood R, Rao S, Ranganathan P, Sanjeeviah RC, Vijayakumar M, Kumar KV, Montgomery EA (2009). Genomewide mRNA profiling of esophageal squamous cell carcinoma for identification of cancer biomarkers. Cancer Biol Ther.

