# Supplementary material for: Chronic exposure to cigarette smoke leads to activation of p21 (RAC1)-activated kinase 6 (PAK6) in non-small cell lung cancer cells
Source: Oncotarget. 2016 Aug 16;7(38):61229–45. doi: 10.18632/oncotarget.11310 (PMC5308647; doi:10.18632/oncotarget.11310)
Supplement: Supplementary file 1 [file oncotarget-07-61229-s001.pdf]

Chronic exposure to cigarette smoke leads to activation of p21 (RAC1)-activated kinase 6 (PAK6) in non-small cell lung cancer cells

Supplementary Materials

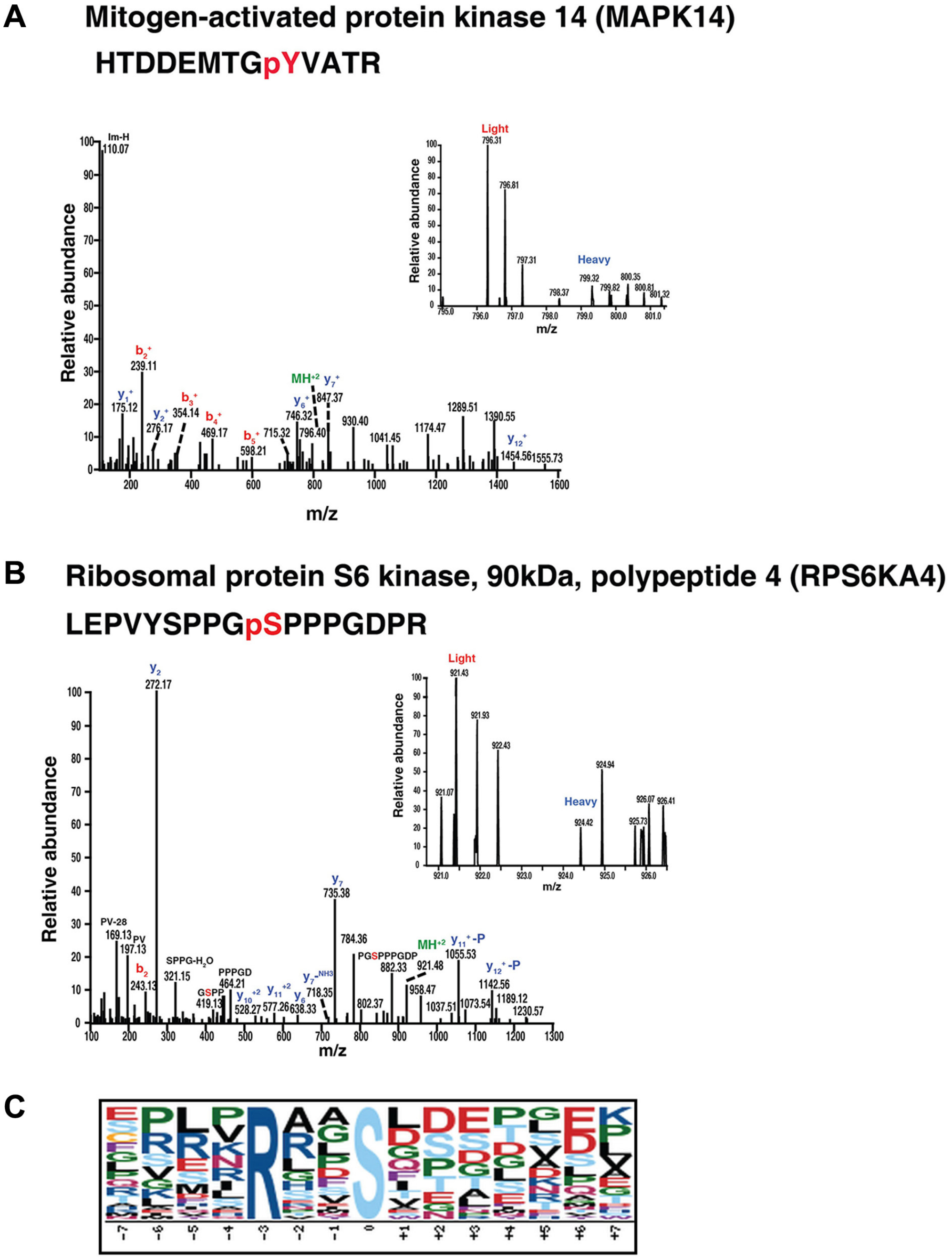

Supplementary Figure S1: Representative MS and MS/MS spectra of hyperphosphorylated peptide of (A) mitogen-activated protein kinase 14 in smoke treated cells and (B) of Ribosomal protein S6 kinase, 90kDa, polypeptide 4. (C) RxxS (consensus motif for AKT) were enriched in hyperphosphorylated site in cigarette smoke treated cells.

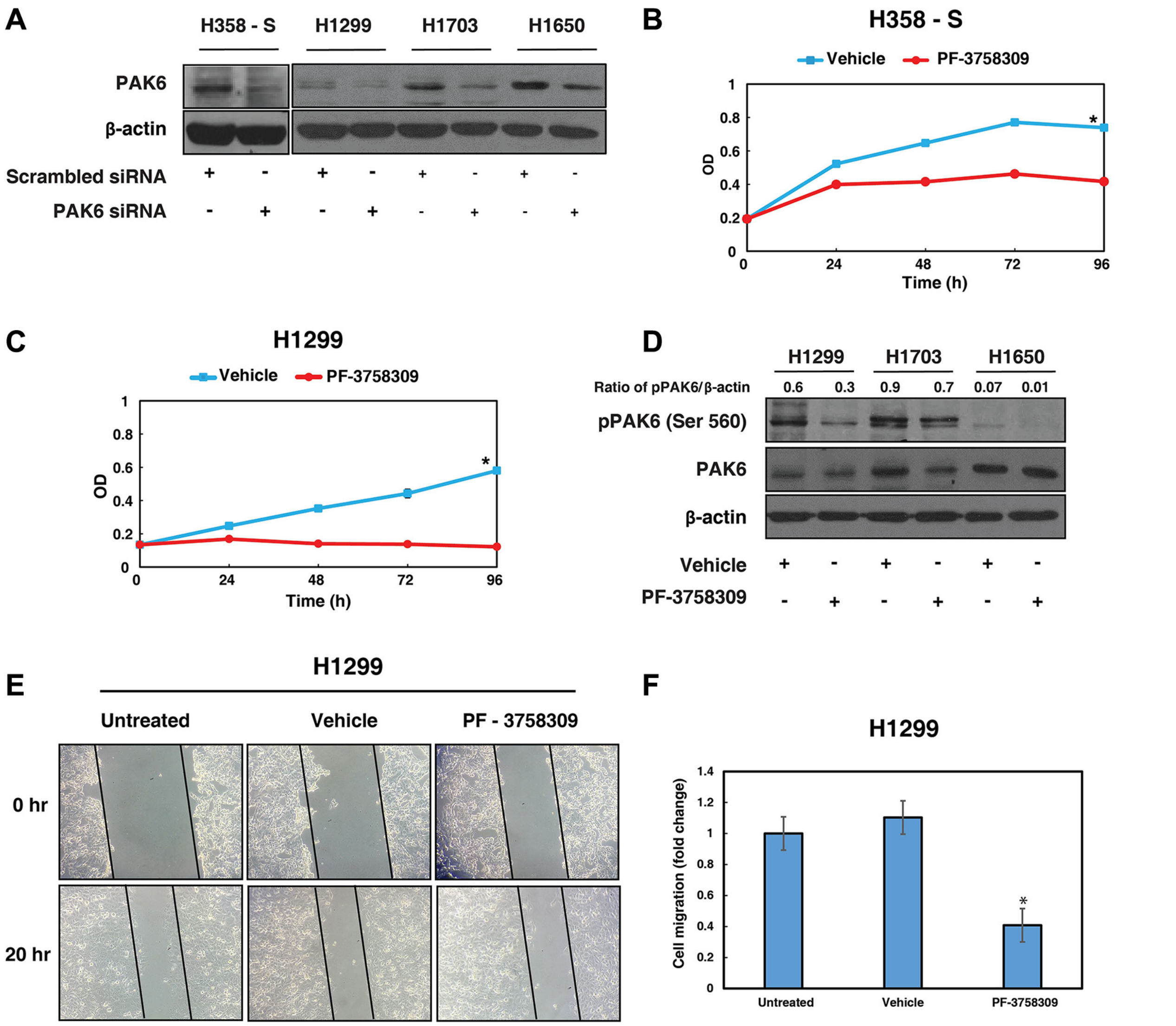

Supplementary Figure S2: (**A**) Western blot analysis of PAK6 in H358-S and three NSCLC cell lines upon siRNA mediated knockdown of PAK6 (PAK6 siRNA) or control (scrambled siRNA) (**B**) Proliferation curve of H358-S cells following PF-3758309 (4 μM) treatment (**C**) Proliferation curve of H1299 cells following PF-3758309 (4 μM) treatment (**D**) Western blot analysis of total PAK6 and pPAK6 (S560) in NSCLC cells upon treatment with PAK6 inhibitor PF-3758309 or control (vehicle). (**E**) Wound migration assays were carried out using H1299 cells with or without PF-3758309. Representative photographs are shown from 0 and 20 hrs. (**F**) Distance migrated by cells was calculated and represented as bar graph. *$p < 0.05$.

Supplementary Table S1: List of all phosphopeptides identified in H358-P and H358-S cells using Sequest and Mascot search algorithms. The table lists peptide, gi accessions, gene symbol, gene ID, protein, modifications, PhosphoRS Site Probabilities, H358-smoke/H358-P , *q*-value, *e* value, missed cleavages, change, m/z, MH+, Δ M, PhosphoSite (Peptide), PhosphoSite (Protein), Phosphowindow, Ion score, XCorr and Spectrum file. See Supplementary_Table_S1
